# The Joint Contribution of Activation and Inhibition in Moderating Carryover Effects of Anger on Social Judgment

**DOI:** 10.3389/fpsyg.2017.01435

**Published:** 2017-09-25

**Authors:** Marina Fiori, Vera Shuman

**Affiliations:** ^1^Department of Organizational Behavior, Faculty of Business and Economics, University of Lausanne Lausanne, Switzerland; ^2^Department of Economics, University of Lausanne Lausanne, Switzerland

**Keywords:** attention, inhibition, activation, anger, cooperative suppression, self-regulation failure, carryover effects, resources depletion

## Abstract

Carryover effects of emotions that lead to biases in social judgments are commonly observed. We suggest that such effects may be influenced by the ability to engage or disengage attention from emotional stimuli. We assessed the ability to activate and inhibit attention to anger stimuli, experimentally induced anger in a demanding task, and measured social judgment toward an ambiguous target. Results show that higher activation and higher inhibition of anger-related information predicted more biased evaluations of the ambiguous target when individuals were experiencing anger, but not in an emotionally neutral condition. Interestingly, the effect of activation and inhibition in the anger condition emerged only when such variables were entered simultaneously in the regression model, indicating that they had an *additive effect* in predicting carryover effects of anger on social judgement. Results are consistent with a cooperative suppression effect (Conger, 1974) of activation and inhibition and may be explained by either an increased accessibility of anger-related cues leading to more biased social judgments, or by an instance in which being good at engaging in and disengaging attention from emotional cues might have depleted participants’ resources making carryover effects of anger more likely to occur. Ultimately, the finding highlight that individual differences in attentional processes are important moderators for carryover effects of emotions.

## Introduction

It is not unusual to have a bad day at work and end up blaming one’s partner at home for something that was nobody’s fault. Are some people better than others at letting go of negative events? Which emotional processes influence how people perceive others after experiencing anger? This study seeks to answer these questions by examining how incidental anger, that is, anger triggered by a reason unrelated to the current event, in combination with individuals’ engagement in or disengagement from anger-related stimuli, biases social judgments.

Anger is a pervasive emotion in everyday life, often requires some form of regulation, and may have quite different effects depending on the circumstances. Sometimes feeling angry may boost resource mobilization in a profitable way, for example by promoting approach tendencies that remove obstacles toward the desired goal (Carver and Harmon-Jones, 2009). At other times it may interfere with performance by drawing attention away from the task or by influencing judgments and behavior regardless of the true source of anger (Lerner and Tiedens, 2006).

**FIGURE 1:**
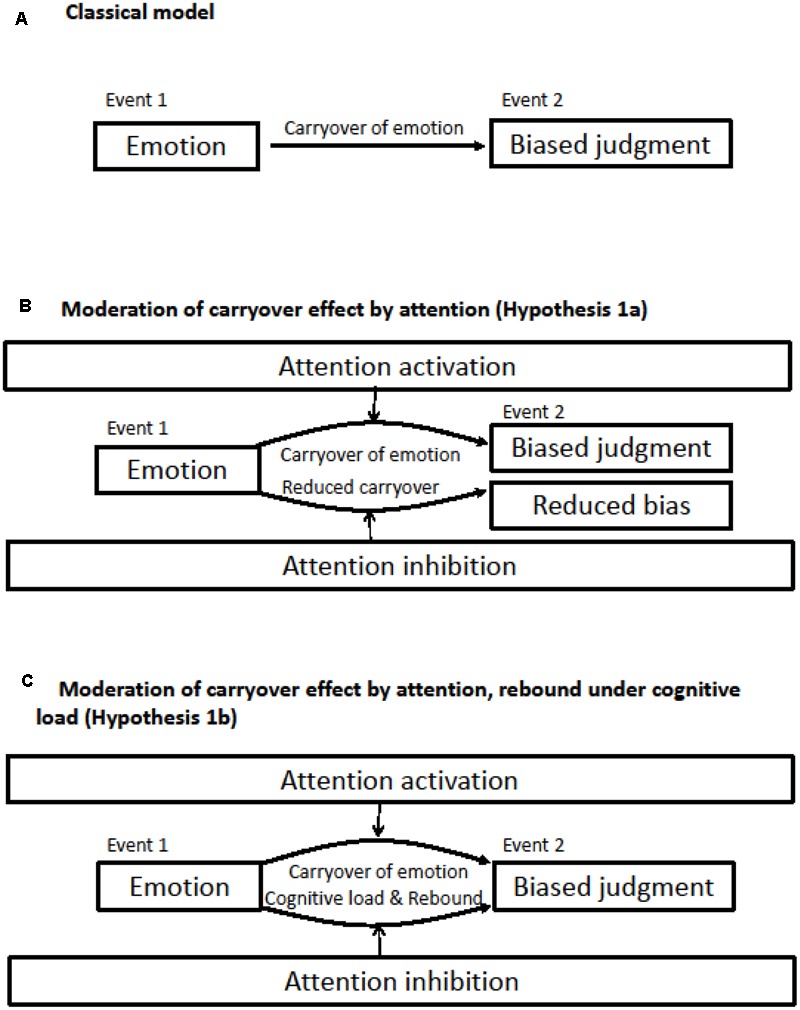
Illustration of the study’s hypotheses. **(A)** The typical carryover effect of emotion on judgment; **(B)** activation accentuates and inhibition reduces carryover effects of emotion on judgment; activation and inhibition both accentuates carryover effects of emotion on judgment.

Emotions often guide information processing and behavior in an adaptive way, but not always. Throughout evolution, the active interpretation of emotional signals has increased the chances of survival (Cosmides and Tooby, 1992). However, relying on emotions when decisions have nothing to do with the true cause of one’s emotions may bias information processing (Forgas, 1995; **Figure 1A**). For example, happiness and sadness increase attention to affect-consistent information and result in judging others more positively or negatively, respectively (Forgas and Bower, 1987). Similarly, individuals who experience anger provide more punitive judgments of unrelated targets (Lerner et al., 1998). The effect of emotions on decision-making is a well-established phenomenon (Forgas, 1991). Mood congruent effects consist of judgments biased toward the current mood state. According to the mood-as-information theory current mood serves, implicitly, as a source of information for the judgment at hand (Schwarz and Clore, 1983). An individual typically uses the appraisals associated with affective states as information about a current situation, but the appraisals may also be carried over to an unrelated event and bias information processing (e.g., Lerner and Tiedens, 2006). Another account that explains bias is the automatic activation of semantic networks that bring emotion congruent material to mind and influence attention and interpretations (Bower, 1981).

Individuals may differ from each other in how much they carry over their emotions to other events. They can regulate their affective reactions by increasing and decreasing how much attention they pay to emotional stimuli. Selecting certain aspects of the situation through one’s focus of attention may be used as a regulatory strategy to prolong a desirable emotional state, or to change an undesired state (Wadlinger and Isaacowitz, 2011). Individual differences in the ability to disengage attention from stimuli are associated with reduced negative affect already in infants (Rothbart et al., 1992). Young children actively manage their emotions by avoiding sensory input from negative emotional stimuli, for example, by closing their eyes. Older children additionally learn to redirect their attention internally (e.g., by thinking about something else; see Thompson, 1994). In adults, too, the ability to disengage attention has been associated with reduced emotional reactions (Compton, 2000). In contrast, ruminating about anger related events prolongs anger (Rusting and Nolen-Hoeksema, 1998). Individuals can use engagement of attention to prolong emotions, and thereby increase a carryover of emotions to unrelated situations. They can use disengagement of attention to end emotions, and thereby prevent a carryover of emotions to unrelated situations.

However, being able to disengage attention from emotional stimuli may not always reduce the carryover of emotions to new situations, and therefore reduce bias in subsequent information processing. For example, individuals high in negative affect who were asked to suppress negative feelings while recalling negative events experienced more negative emotions than individuals who had not received the instruction to suppress feelings (Dalgleish et al., 2009). Trying to suppress happiness or sadness in a cognitively demanding situation may lead to the opposite result. In ironic process theory (Wegner, 1994), individuals who were instructed to suppress thoughts about a ‘white bear’ express more thoughts about it as compared to individuals who had not received instructions to suppress thoughts. Eventually it was demonstrated that the attempt to avoid certain thoughts/information may cause such thoughts to be more accessible, producing a backfire effect that was documented in the literature as rebound or ironic effect (Wegner, 1994, 2009). It was hypothesized that two processes are engaged during thought suppression: an automatic process or ‘monitoring system,’ which searches for instances of the unwanted thought and does not require cognitive resources to run; and a controlled process or ‘operating system,’ which is activated any time the unwanted thought is found with the purpose of replacing it with something else. Increased accessibility of the suppressed thought may occur: (a) at the moment in which the person is attempting to suppress it, in case cognitive load weakens the operating system; or (b) after suppression successfully occurred, when the operating system is relaxed or terminated. In the latter case, also known as ‘the post-suppression rebound effect’ (Wegner, 1994), individuals may be effective in suppressing certain thoughts until control is exerted. As soon as control is released, the rebound effect may appear.

Clark et al. (1991) tested the rebound hypothesis using this experimental design: Participants listened to a taped story and verbalized their thoughts during 2 time periods. For the first recall, one group was told to express any thought that came to mind except thoughts referring to the tape (suppression condition); another group was told to express any thought (control condition); and the third group was told to express any thought including those referring to the tape (control condition). For the second recall, all three groups were asked to think about any thought that came to mind. The authors did not find increased accessibility of thoughts related to the tape in the suppression group during the first recall. However, participants in the suppression condition displayed increased accessibility of thoughts related to the tape during the second recall, when they did not have specific suppression instructions. This may be explained by the fact that the monitoring system may continue to work even after the operating system has stopped and lead to hypersensitivity to the suppressed thought. The result is the paradoxical effect that suppressed thoughts end up working as retrieval cues.

These findings suggest that inhibiting the processing of anger stimuli may later result in increased reactivity in cognitively demanding anger arousing events, subsequently biasing information processing.

Lord and Levy (1994) suggested that activation and inhibition are fundamental processes for regulating behavior. Activation ensures the consideration of goal-relevant information, whereas inhibition prevents goal-irrelevant information from interfering with task execution. Inhibition is defined as «the stopping or overriding of a mental process, in whole or in part, with or without intention. The mental process so influenced might be selective attention or memory retrieval or a host of other cognitive processes.» (MacLeod, 2007, p. 5). In this paper, we examine how the activation and inhibition of attention to anger stimuli influences the carryover of anger as reflected in biased social information processing after experiencing incidental anger.

**FIGURE 2:**
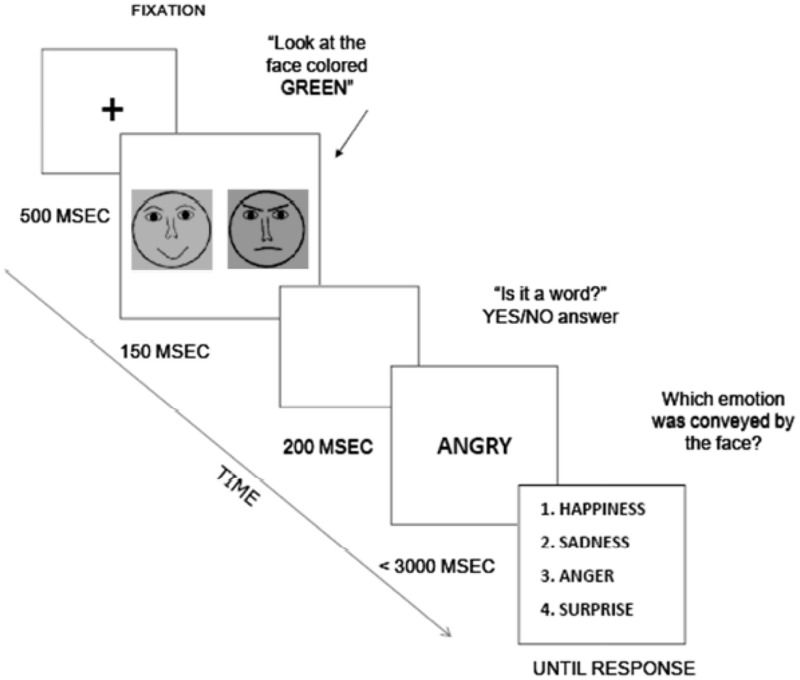
Illustration of the attention task employed in the study.

In the present research, activation and inhibition were measured employing a task that presents similarities with affective negative priming in the emotion literature (e.g., Joormann and Gotlib, 2010) and semantic priming in the cognitive literature (e.g., Ortells et al., 2001). The task consisted of a series of two consecutive trials (**Figure 2**). In the prime trial, two diagrams representing faces with different emotions (anger, sadness, and happiness) were presented next to each other in two different colors (green and blue), and participants were instructed to pay attention to only the green face. In the test trial, a letter string appeared and participants had to indicate whether the letter string was a word, as in a typical lexical decision task (LDT). Participants responded by pressing a letter on the computer keyboard designated as ‘yes’ or ‘no.’ The word could either be related in affective tone to the face participants were instructed to look at (the target), to the face participants were instructed to ignore (the distractor), or unrelated to both (the control condition). Response Time (RT) to the LDT was used to calculate activation and inhibition for three emotion categories: anger, sadness, and happiness. In every trial, after the LDT participants performed a categorization task. They indicated, among a list of four options, which emotion was conveyed by the green face. This latter task was introduced to ensure that participants paid attention to the expression conveyed by the prime.

Performance in the LDT involving the judgment of a word related to the target was expected to be facilitated, both in terms of speed of processing and in terms of correct answering. According to the associative network model (Bower, 1981) information is stored in nodes that are related by semantic and affective meaning. When a node is activated it also activates other connected nodes. In the case of the present task, the prime should activate a network of similar valence information that would increase the accessibility of related nodes and, consequently, foster correct responses.

Instead of being facilitated, performance involving the judgment of a word related to the distractor was expected to be impaired. Paying attention to a stimulus that was previously dismissed from attention should interfere with task execution, making information processing slower and performance more challenging. Explanations of inhibition effects include impaired retrieval in processing information previously categorized as distractors (Neill, 1977; Tipper, 1985, 2001; Yee, 1991; Houghton and Tipper, 1994) and conflict between two possible responses: ‘select it’ because it is the current object of evaluation, or ‘ignore it’ because it was first introduced as a distractor (Neill and Valdes, 1992). Both explanations support the presence of inhibitory mechanisms that suppress responses–either memory retrieval or attentional focus–suggesting that inhibitory processes play a role in RT.

A study that employed the same paradigm discussed above investigated the effect of individual differences in inhibition on task performance characterized by emotional involvement (Moon and Lord, 2006). The criterion measure was performance on two tasks requiring fast emotion regulation: a scrambled sentence task, and an editing task, both characterized by time pressure, and high emotional content that, if not regulated, could interfere with performance. Results showed that difference in RTs to the distractor-consistent stimulus predicted performance.

Another study (Fiori and Antonakis, 2012) showed that performance in the LDT requiring activation and inhibition of emotion information was predicted by the fluid component of intelligence, in particular as measured by Cattell’s Culture Fair Test (Cattell et al., 1973). Interestingly, the only personality factor that predicted performance was openness to experience, which is the personality trait more strongly associated with general intelligence, supporting the idea that activation and inhibition of emotion information pertain to the intelligence, more than the personality, domain.

Although attentional bias toward specific emotions, such as fear, has been widely studied with reaction time paradigms, such as the stroop task (e.g., Fox et al., 2002), fewer studies have investigated the role of individual differences in attentional processes as predictors of behavior outcomes. Several of the studies employed self-report measures of anger, rather than performance-based measures (e.g., Denson et al., 2012). One study showed that individuals low, as compared to high, in stroop task performance designed to measure general attentional control judged moral behavior more severely after being primed with disgust.

## The Present Research

We investigated individual differences in anger-specific activation and inhibition as moderators of the relationship between anger and social judgment. More specifically, the study was designed to analyze the role of individual differences in the activation and inhibition of anger-related information in a situation in which anger was not related to the task at hand and to observe the effect on performance. In particular, the reaction to anger was analyzed with respect to its influence on forming impressions of an ambiguous character that is completely unrelated to the experience of anger and that could be perceived as more or less hostile.

We hypothesized that individuals higher in activation to anger stimuli would show more bias in a social judgment task after an anger manipulation, and that individuals higher in inhibition would show less bias in this situation because of their capacity to disengage from anger stimuli (Hypothesis 1a, **Figure 1B**). Given that the literature on ironic processes has suggested that suppressing emotions may lead to an ironic increase in such feelings (Dalgleish et al., 2009) we also posited an alternative that inhibition may increase biases in judgments because of ironic processes (Hypothesis 1b, **Figure 1C**). A relatively demanding task during emotion induction allowed for the post-suppression rebound effect.

## Materials and Methods

### Design and Procedure

To examine these hypotheses, we used three tasks. The first task measured the ability to activate and inhibit attention to anger related stimuli. Reactivity to anger induction was then assessed with an anger manipulation. Finally, potential biases in information processing were assessed in a social judgment task. The study had a 2 × 2 mixed design with attention to anger-related information (activation and inhibition) measured within subjects and emotion induction (anger/control) manipulated between subjects. The dependent variable was the extent of negative trait ratings in the social judgment task.

### Participants

Ninety participants (46 females) were recruited from a US Midwestern University subject-pool in exchange for partial course credit. During the funnel debriefing, two participants seemed to know the purpose of the experiment from the beginning. Furthermore, data for three participants were lost due to error in administration of Eprime. These five participants were dropped from the analyses. In the end 85 out of 90 were retained for the statistical analysis, with 45 participants assigned to the anger condition (25 females) and 40 to the control condition (20 females). This study was carried out in accordance with the recommendations of the ‘Office for the Protection of Research Subjects (OPRS) of the University of Illinois at Chicago’ with written informed consent from all subjects. All subjects gave written informed consent in accordance with the Declaration of Helsinki. The protocol was approved by the ‘IRB of the OPRS.’ Participants were 53% females and 47% males. The mean age was 19.13 (*SD* = 2.05), with age ranging between 17 and 28. The composition of the sample was: Asians (40.4%), Whites (29.2%), Hispanics (16.9%), and African-Americans (7.9%). A small percentage of individuals (5.6%) did not indicate their race.

### Measures

#### Attention Task

The procedure of the task was the same employed in other studies (Moon and Lord, 2006; Fiori and Antonakis, 2012). The task consisted of 96 consecutive trials. In each trial, 2 diagrams representing emotional facial expressions (anger, sadness, and happiness) were presented side by side, one in green and one in blue (**Figure 1**). These basic emotions were selected because they were clearly identified by participants in previous research (Shaver et al., 1987). Face diagrams were created according to emotion expression key features indicated by Ekman and Friesen. In each trial two faces, measuring 2 inches × 2 inches each, were displayed side by side for 200 ms. Participants were instructed to attend to the green face, which changed side randomly across trials for each participant, so that participants had to look at both facial expressions to identify the one they had to attend to. Then, a letter string appeared for 3 s and participants indicated whether it was a word, as in a typical LDT. The letter string was either congruent in valence with the target face (for example, a green angry face followed by an ‘anger’ word), or congruent with the distractor face (for example, a blue angry face followed by an ‘anger’ word). Letter strings were systematically varied from a list of synonyms of emotional words referring to anger, sadness, happiness, and surprise, the latter being included as a control condition, together with non-words of the same length. To avoid inter-trial effects^1^—the activation of particular emotion information in one trial could affect the processing of emotion information in the next trial—and to ensure that participants paid attention to the expression conveyed by the face and not only its color, after each trial participants performed a categorization task. They indicated the emotion conveyed by the target face from a list of four emotions.

Response Times (RTs) to anger-related words congruent with the angry target face were taken as a measure of activation of attention, with faster RTs indicating higher activation. RTs to anger-related words congruent with the angry distractor face were taken as a measure of inhibition, with slower RTs indicating higher inhibition (for more information on the task, see Moon and Lord, 2006; Fiori and Antonakis, 2012). There is an ongoing debate regarding whether RTs to priming tasks, of the same type as that employed in the current study, should be calculated as difference scores with respect to a control condition or as simple RTs. We calculated difference scores by subtracting RTs in the anger congruent conditions with angry faces as a target or distractor from RTs in the condition in which angry faces (targets or distractors) were followed by happiness, sadness or surprise words (control condition). Using simple RTs produced the same pattern of results (see similar results in Chan et al., 2006). Thus, for the sake of clarity when interpreting results simple RTs were retained, and they are presented in the current paper.

#### Anger Induction

The present study employed a modified version of an anger induction procedure successfully used by Kenworthy et al. (2003) and Pedersen et al. (2000; Study 2). Participants expected to perform a task meant to analyze the influence of sounds on performance: The task consisted of solving 15 anagrams while listening to background sounds. Participants were invited to wear headphones with a microphone. Before participants started reading instructions about the experiment, the experimenter informed them that the system was equipped with an audio-recording system for which their response to the task would be audio-recorded. Anagrams appeared on the screen for 10 s each, followed by a screen prompt for the answer. Participants had 5 s to provide the answer. They were told to solve the anagrams and then say the answer aloud. In case they did not know the answer, they were instructed to say ‘I don’t know’ out loud.

There were three factors specifically related to the induction of anger. First, anagrams were very difficult to solve, including words such as pandemonium. Second, the sound in the background was quite loud (60 db) and irritating (it was a passage from Stravinsky’s ‘Rite of Spring’). Third, after the 4th, 8th, and 12th anagram participants were interrupted by a message popping up on the screen lamenting that the participant needed to speak louder. The tone and intensity of the message increased throughout the task and displayed more and more annoyance and irritation. In the control condition participants solved much easier anagrams, including words such as time, they listened to a neutral background sound – the noise of light traffic recorded in downtown Chicago on a weekday, which was also played softer, and the interruption that popped up simply informed participants how many anagrams remained until the end of the task.

An anger manipulation of this kind was chosen because it was related to a sort of incidental anger, rather than anger directed to a social target, such as when the experimenter explicitly mocks the participant for negative performance. For the present study, the latter kind of anger induction was excluded to avoid any unexpected priming of thoughts related to ‘social’ aspects of anger, which could have confounded results of the subsequent impression formation task. Furthermore, this anger manipulation has been proven to induce anger and frustration more than other emotions (Kenworthy et al., 2003). No emotion manipulation elicits only one emotion. However, because some emotions may have different effects on decision-making, it is always important to ensure that the manipulation works in the expected direction. The manipulation procedure lasted approximately 5 min.

#### Social Judgment Task

Participants read a vignette taken from Srull and Wyer (1979) regarding the description of a day in the life of Donald, the protagonist. As pointed out by Otten and Stapel (2007), Srull and Wyer’s (1979) task has been successfully used in several studies, such as Bargh and Pietromonaco (1982) and Devine (1989), to demonstrate the effects of priming on anger and aggression.

The vignette includes 20 sentences containing 5 somewhat hostile but ambiguous behaviors (e.g., not letting a salesperson enter an apartment). Participants the rated positive and negative traits of the target person on a unipolar 9-point Likert scale. The average rating for negative traits (hostile, unfriendly, dislikable, boring, selfish, and narrow-minded) was the study’s dependent variable.

### Manipulation Checks

After the emotion manipulation (anagrams task), participants filled out a questionnaire about their current emotional state. Becoming aware of one’s emotional state may reduce the impact of emotions on subsequent activities, especially when such activities are unrelated to the emotion felt (Schwarz and Clore, 1983), and labeling emotional states may reduce their impact on judgment (Keltner et al., 1993). For these reasons a subtle manipulation check was used. Instead of directly asking participants whether they were irritated, depressed, or relaxed, they were asked to rate how they found the music (irritating, relaxing, and depressing), with questions such as ‘Did you find the music irritating.’ Responses were reported on a Likert-type scale from 1 = not at all to 9 = very much. Furthermore, questions related to the emotional state, in particular ratings of irritation and sadness, were mixed with a series of filler questions related to the task itself, such as ‘Do you like working/studying with music in the background?’ A funnel debriefing was employed to check whether participants had any suspicion about the anger manipulation: It started with broad questions about the purpose of the study and proceeded to very specific questions referring to participants’ understanding of the experimental conditions/design. The vast majority of individuals had absolutely no clue about the purpose of the experiment and about the fact that anger was induced on purpose. To the question regarding how they felt during the anagram task, most participants responded “I was mad.”

## Results

The anger induction was effective: subjects judged music/sound in the background to be more irritating in the anger (*M* = 5.69, *SD* = 3.22) than in the control condition (*M* = 4.28, *SD* = 3.02), *F*(1,84) = 4.2, *p* < 0.05. The anger (*M* = 3.15, *SD* = 2.41) and control condition (*M* = 3.92 *SD* = 2.41) did not differ in how much participants liked the music/sound in the background.

Correlations of the study’s variables are reported in **Table 1**. Data were analyzed using STATA version 14 (StataCorp, 2015) and hypothesis tests involved running hierarchical regressions with robust variance estimation in which the study variables were entered in sequential steps to predict negative trait ratings. This analytical strategy was chosen to deal with violations in the assumption of linear regression, such as normality of residuals. Only correct answers in the attention task were retained for the analysis (84% of all answers), and participants who provided less than 50% of correct answers were excluded from the analysis (8 individuals). RTs faster and slower than 2 standard deviations from the mean were trimmed.^2^

**Table 1:** Correlation of the study variables.

Variable	*N*	Mean	Std. Dev.	Sex (*F* = 1)	Manip. (anger = l)	Irritation	Inhibition	Activation	Neg. ratings
Sex (*F* = l)	85	0.48	0.50	1.00					
Manip. (anger = l)	84	0.54	0.50	-0.05	1.00				
Irritation	84	5.03	3.19	-0.13	0.22^∗^	1.00			
Inhibition	75	1142.41	289.05	-0.12	0.06	0.00	1.00		
Activation	75	1179.47	303.55	0.10	0.04	0.09	0.47^∗^	1.00	
Negative ratings	75	1179.47	303.55	0.03	0.27^∗^	0.22^∗^	0.02	-0.01	1.00

Because activation and inhibition were significantly correlated with each other and to fully understand the contribution of each of these predictors, we conducted two hierarchical regressions in which we tested the effect of each predictor and the interaction with the anger manipulation. Then we conducted a third hierarchical regression in which both predictors and their interaction terms were entered simultaneously. In all the analyses, we controlled for gender because some studies have shown that males and females differ with respect to how they describe emotional experience (Barrett et al., 2000). Activation and inhibition were centered before conducting the regression analysis. Results are presented in **Table 2**.

**Table 2A:** Results of the hierarchical regression involving only activation and the interaction with the anger manipulation term as predictors of social judgment (negative ratings of Donald).

	Step 1				Step 2				Step 3				*F*	*p*
	St. Coef.	SE (Robust)	*t*	*p*	St. Coef.	SE (Robust)	*t*	*p*	St. Coef.	SE (Robust)|	*t*	*p*		
Sex (*F* = l)	0.14	0.34	1.29	0.20	0.14	0.34	1.27	0.21	0.13	0.33	1.16	0.25	3.95	0.02
Manip. (anger = l)	0.26	0.34	2.30	0.02	0.26	0.33	2.30	0.02	0.98	1.26	2.30	0.02		
Activation					0.005	0.00	0.05	0.96	0.16	0.00	1.85	0.07	0.00	0.96
Activation × Manip.									-0.77	0.00	-1.78	0.08	3.18	0.08
Constant									5.77	0.28	20.40	0.00		
R-square				0.08				0.08				0.12		

**Table 2B:** Results of the hierarchical regression involving only inhibition and the interaction with the anger manipulation term as predictors of social judgment (negative ratings of Donald).

	Step 1				Step 2				Step 3				*F*	*p*
	St. Coef.	SE (Robust)	*t*	*P*	St. Coef.	SE (Robust)	*t*	*p*	St. Coef.	SE (Robust)	*t*	*p*		
Sex (*F* = l)	0.11	0.35	0.95	0.35	0.12	0.36	0.99	0.32	312.00	0.38	1.07	0.29	2.44	0.09
Manip. (anger = l)	0.23	0.35	1.98	0.05	0.22	0.36	1.92	0.06	-0.29	1.48	-0.60	0.55		
Inhibition					0.06	0.00	0.46	0.65	-0.05	0.00	-0.26	0.80	0.21	0.65
Inhib. × Manip.									0.55	0.00	1.05	0.30	1.11	0.30
Constant									5.76	0.3	18.91	0.00		
R-square				0.06				0.06				0.08		

**Table 2C:** Results of the hierarchical regression involving both activation and inhibition and the interaction with the anger manipulation term entered simultaneously as predictors of social judgment (negative ratings of Donald).

	Step 1				Step 2				Step 3				*F*	*p*
	St. Coef.	SE (Robust)	*t*	*p*	St. Coef.	SE (Robust)	*t*	*p*	St. Coef.	SE (Robust)	*t*	*p*		
Sex (*F* = l)	0.15	0.33	1.38	0.19	0.17	0.36	1.44	0.15	0.20	0.35	1.73	0.09	4.10	0.02
Manip. (anger = l)	0.27	0.33	2.37	0.02	0.27	0.34	2.31	0.02	0.29	1.59	0.54	0.60		
Activation					-0.04	0.00	-0.34	0.74	0.19	0.00	1.74	0.09	0.23	0.80
Inhibition					0.11	0.00	0.67	0.50	-0.11	0.00	-0.55	0.59		
Activation × Manip.									-1.37	0.00	-2.96	0.01	5.38	0.02
Inhib. × Manip.									1.35	0.00	2.50	0.02		
Constant									5.66	0.29	19.65	0.00		
R-square				0.09				0.10				0.21		

**FIGURE 3:**
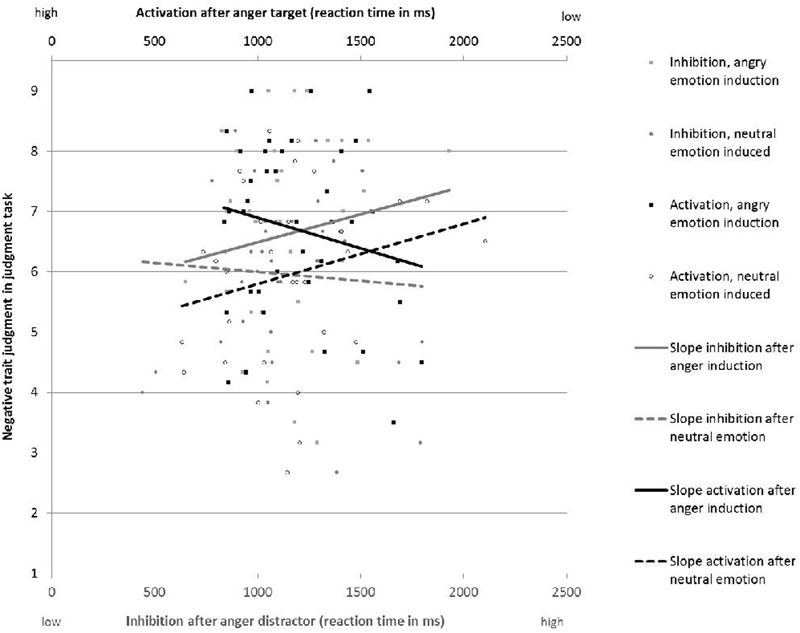
Judgments of a target (*y*-axis) tend to be more negative after an anger induction. The greater the activation of attention to anger related stimuli (top *x*-axis) the greater the negative judgment; the greater the inhibition of attention to anger stimuli (bottom *x*-axis) the greater the negative judgment. Note that the RT scale for activation is interpreted in the opposite way with respect to inhibition: shorter RT indicates stronger activation.

A comparison of the regression models shows that activation and inhibition and the interaction with the anger manipulation term do not predict negative ratings in the social judgment task when taken individually (**Tables 2A,B**). However, their effect becomes significant once they are entered simultaneously in the regression model (**Table 2C**). To plot the significant interactions (**Figure 3**), we computed separate regression lines for each emotion condition (anger/neutral). In the anger condition, the model was significant, *F*(1,36) = 3.25, *p* = 0.05, and both activation (β = -0.40, *t* = 2.21, *p* < 0.05) and inhibition (β = 0.40, *t* = 2.20, *p* < 0.05) were significant predictors. Individuals in the anger condition rated the target person particularly negatively when they had shown higher activation in the attention task (e.g., shorter reaction times after anger target cues). Similarly, individuals in the anger condition rated the target person particularly harshly when they had shown higher inhibition in the attention task (=longer reaction times after anger distractor cues; **Figure 3**). In the neutral condition, the model was not significant *F*(1,31) = 1.17, *p* > 0.05. Overall, the model explained 21% of the total variance in negative ratings.

Overall results were aligned with the hypothesis that both higher activation and higher inhibition would lead to more negative ratings (hypothesis 1b). However, the finding that the effect of activation and inhibition in the anger condition was significant only when *both* variables were entered *simultaneously* was unexpected. To further explore their association we tested the interaction between activation and inhibition (and the experimental condition, in a 3-way interaction) as a predictor of negative ratings. Results revealed no significant effect, indicating that the contribution of activation and inhibition in predicting negative ratings is in fact additive, and not interactive.

Finally, because RTs were also collected for happiness and sadness activation and inhibition, we tested whether they yielded any significant effect. Indeed, we hypothesized anger-specific effects. Remarkably, activation and inhibition of happiness and sadness related information did not, individually or jointly, predict social judgment. This provides supporting evidence that our effects are specific to the emotion that was applicable to the social judgment, namely anger, and that this effect is not a generic effect of activation and inhibition.

## Discussion

The purpose of the present study was to investigate how individual differences in the attentional engagement to and disengagement from anger related stimuli influence processing biases after experiencing anger. Our study replicates previous research (Lerner and Tiedens, 2006) showing that incidental anger results in biased social information processing. After anger induction, individuals judged another person in an unrelated situation more negatively.

Moreover, our results add to the literature by providing two important contributions. First, we show that carryover effects are more pronounced in individuals with higher activation and higher inhibition of attention to anger related stimuli. The more individuals engaged attention to anger stimuli, the more they carried their anger over to an unrelated situation resulting in more biased social judgments. We found similar effects for individuals with higher inhibition of attention to anger stimuli, who demonstrated stronger biases after the anger induction. Second, we also show that the effect of activation and inhibition emerged only when these attentional processes additively contributed to performance. Indeed, when activation and inhibition were taken individually they did not moderate the carryover effects of anger on social judgment.

The case in which the predictive power of two variables significantly increases once the variables are entered into the regression model at the same time is known in the literature as a suppression effect^3^ (Conger, 1974). In particular, in the case of a cooperative or reciprocal suppression (Cohen and Cohen, 1975; Paulhus et al., 2004) the direction of the association between the two predicting variables and the outcome is opposite in sign. This as is also the case for activation, the interaction term of which is negatively associated with negative ratings, and inhibition, the interaction term of which is positively associated with negative ratings. Entering the two interaction terms at the same time allows partitioning out the shared variance between the two variables, and lets each unique variance predict the outcome in opposite directions (for similar cases discussed in the literature see Verona et al., 2005; Geraghty et al., 2010).

Our results point out that in order to observe carryover effects of anger on social judgment the simultaneous effect of both activation and inhibition must be taken into account. Interestingly, the joint effect of these two variables was additive rather than interactive, as shown by the analysis in which the interaction between these variables and the experimental condition was not significant. In other words, both variables accentuated the carryover effect of anger, and it seems as if each of them taken individually was not sufficient to bias social perception; both were necessary for the effect to emerge.

Two competing explanations may account for the results. The first explanation refers to the effect of increased accessibility of previously processed emotional cues. In the present research, some participants were better than others at paying attention to and at ignoring anger-related cues when asked to do so. However, when no specific instructions were provided about what to do with similar cues and in a different context, the same participants displayed higher sensitivity to this type of information, which biased their social perception. It seems as if the effort to activate and inhibit anger related information was successful during the attention task, but at the cost of increasing the availability of the same type of information later. The results are congruent with a post-suppression rebound effect (Wegner, 1994), although in the present case the increased accessibility of the previously suppressed emotion information was exacerbated in individuals who were also good at activating the same type of information.

An alternative explanation that is also plausible in explaining results especially in light of the unexpected *joint* effect of activation and inhibition is that participants’ resources might have been depleted by both the anger manipulation and the attention task—which were rather cognitively demanding—a situation that made it likely for any attempt to regulate the interplay of activation and inhibition in subsequent tasks to fail (Baumeister et al., 2007). Indeed, our results might describe a case of self-regulation failure in which being good at engaging and disengaging attention to emotional cues might have depleted participants’ resources making it more likely that carryover effects of anger would occur.

It is interesting to notice that the correlation between activation and inhibition was moderately high and positive in sign, indicating that on average people who were better (e.g., quicker) at activating anger related information were also worse (e.g., quicker) at inhibiting the same type of information. However, some individuals showed a pattern of being good at both activating and inhibiting information, and those were also the individuals who were more prone to carryover effects of anger on social judgment. Indeed, individuals who possess the cognitively and emotionally taxing combination of high activation and high inhibition might be exposed to more rapid consumption of self-regulatory resources, with its related potential side effects (Wagner and Heatherton, 2014), including the undesirable carryover effects of emotions. Further research may clarify which account better explains this type of effect.

Although we treated activation and inhibition as two separate individual characteristics, we cannot rule out the possibility that they tap into the same underlying mechanism, namely sensitivity to emotion cues. Activation and inhibition certainly share the same basic attentional resources that depend on the central executive system. At the same time, it seems as if activation and inhibition possess distinct features because they simultaneously and uniquely contribute to the carryover effect of anger on social judgment. In fact, when their shared variance is ‘suppressed’ by entering the two interaction term predictors simultaneously, they only predict negative ratings. Further research should investigate whether activation and inhibition may be conceived as tapping into the same or distinct cognitive processes, and under which conditions they may contribute individually or jointly to behavioral outcomes.

Another important topic for future research is to clarify whether differences in engaged and disengaged attention are stable over time. Similar to previous research, we assessed attentional processes in one task, and examined their influence on an unrelated task. Although activation and inhibition of attention has been treated as an individual difference variable in previous research (e.g., Compton, 2000; Moon and Lord, 2006), it is currently unknown how stable differences in this ability are over time. For example, it is possible that attention engagement and disengagement varies from day to day, and that training can influence their development (Wadlinger and Isaacowitz, 2011).

## Implications

The results have several applications. Perhaps the most important is that activation and inhibition should be investigated together to allow for testing potential additive effects. Omitting either one may lead to misinterpreting the results, such as finding a lack of significant effect, when in fact such an effect may emerge only if analyzed together with complementary predictors.

The carryover effect of anger was less pronounced for individuals who were medium and low on activation and inhibition, showing that being less extreme in these two characteristics led to less biased social perception. In this respect, Aristotle’s definition of virtue as standing in the middle (also used by the Latins: *in medio stat virtus*) highlights the implications of our findings well. We hypothesized that being effective at both activating and inhibiting emotion information might have significantly taxed cognitive resources leading to undesirable carryover effects of anger.

With respect to the literature on emotion congruent effects (Forgas, 1995; Schwarz and Clore, 2007), the present results show that these effects are moderated by differences in how emotional information is processed. Results show that emotion congruent processing is facilitated in individuals who are able to engage and disengage attention from emotion cues. More generally, stable tendencies may interact with situational emotion stimuli leading to unexpected patterns such as the one we observed.

Becoming aware of the emotion one is feeling during an activity may be the first step to overcoming carryover effects of emotion. For instance, labeling emotional states was found to reduce its impact on judgment (Keltner et al., 1993). Additionally, becoming aware of the cause of an emotional reaction is important to understand whether emotions may be trusted or not during a specific task. Individuals who tend to question why they are feeling a certain emotion may be more capable to regulate emotions according to the situation. The interpretation of results as depending on depletion of resources following highly cognitively demanding tasks suggests that taking a break between two tasks may also be an effective strategy to limit carryover effects of anger.

Finally, the results of this research indicate the importance of considering individual differences in investigating the effect of emotion on behavior/performance. Had predictions been made only based on mood congruency models, we would have expected angry individuals to perceive the target person more negatively than non-angry individuals. Instead, and in addition, results showed that the effect of anger depended on individual differences in activation and inhibition. The effect of anger was boosted for high activation and high inhibition individuals, and nullified for low activation and low inhibition individuals.

In the same line, research on the perception–behavior link (Bargh, 1990; Bargh and Chartrand, 2000) emphasizes the ubiquitous effect of the automatic activation of concepts on congruent behavior. For instance, participants subtly primed with a word referred to rudeness were more likely to behave rudely (Bargh et al., 1996). Given the importance of these findings, it seems worth analyzing whether individuals differ in how they react to emotions caused by external stimuli of which they are not aware. Instead of being treated as noise, individual differences of this kind may reflect the ability to strategically use emotions to better adjust to the situation. For instance, the perception of emotional stimuli may not trigger the same behavior in all individuals. Some people may behave less rudely than others after being primed with rudeness because they integrate emotion with thought and action in a more profitable way, so as to make their behavior more effective with respect to the context.

## Conclusion

We demonstrate the importance of attentional processes for biasing perception under the influence of anger. These processes may function as moderators of emotion carryover effects. Additionally, results show the perils of the joint effect of anger inhibition and activation: being good at both engaging attention to and disengaging attention from anger-related cues may have increased the accessibility of similar emotional information leading to more biased social judgments. As an alternative, being effective at engaging attention to and disengaging attention from anger-related cues may have depleted regulatory resources resulting in stronger carryover effects of anger. These competing explanations merit further investigation.

## Author Contributions

MF designed the study and collected the data. VS provided substantial contribution to the analysis of data and the explanation of results. Both authors were equally involved in the write up of the manuscript.

## Conflict of Interest Statement

The authors declare that the research was conducted in the absence of any commercial or financial relationships that could be construed as a potential conflict of interest.

## Footnotes

1 Some studies demonstrated that there may be inter trial effects or carryover effects following the processing of emotional stimuli. For instance, Sharma and McKenna (2001) found a delay of 11 ms following the processing of negative stimuli at intervals of 1000 ms. In the present study emotion processing was followed by the lexical decision task and then the categorization task, making the inter trial interval at least 4–5 s. This duration makes it unlikely that the inter trial effect could have influenced results.

2 The impact of missing data was examined using structural equation modeling with maximum likelihood estimation for missing data (MLMV). Results were comparable to those reported.

3 We thank Reviewer 1 for suggesting this possibility.
